# The role of CD47 in non-neoplastic diseases

**DOI:** 10.1016/j.heliyon.2023.e22905

**Published:** 2023-11-29

**Authors:** Chao Wang, Ying Feng, Deepali Patel, Hongwei Xie, Yaqing Lv, Hai Zhao

**Affiliations:** a Department of Neurosurgery, the Affiliated Hospital of Qingdao University, No. 16 Jiangsu Road, Qingdao, Shandong, 266005, China; b Department of Emergency, the Affiliated Hospital of Qingdao University, No. 16 Jiangsu Road, Qingdao, Shandong, 266005, China; c School of Medicine, Qingdao University, No. 308 Ningxia Road, Qingdao, Shandong, 266071, China; d Department of Outpatient, the Affiliated Hospital of Qingdao University, No. 16 Jiangsu Road, Qingdao, Shandong, 266005, China

**Keywords:** CD47, SIRPα, Immune modulation, Immunotherapies, Atherosclerosis, Neurological disorders, Autoimmune diseases

## Abstract

CD47 is a 50 kDa five-spanning membrane receptor that plays a crucial role in multiple cellular processes, including myeloid cell activation, neutrophils transmigration, vascular remodeling, leukocyte adhesion and *trans*-endothelial migration. Recent studies have revealed that CD47 is a highly expressed anti-phagocytic signal in several types of cancer, and therefore, blocking of CD47 has shown an effective therapeutic potential in cancer immunotherapy. In addition, CD47 has been found to be involved in a complex interplay with microglia and other types of cells, and increasing evidence indicates that CD47 can be targeted as part of immune modulatory strategies for non-neoplastic diseases as well. In this review, we focus on CD47 and its role in non-neoplastic diseases, including neurological disorders, atherosclerosis and autoimmune diseases. In addition, we discuss the major challenges and potential remedies associated with CD47-SIRPα-based immunotherapies.

## 1 Introduction

**Fig. 1:**
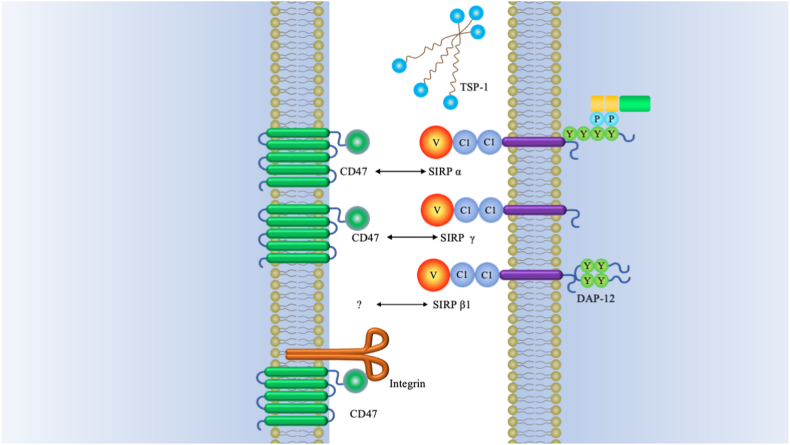
CD47 binding partners Three major groups of ligands are identified to exhibit capacity of binding to CD47, namely, SIRP family, TSP-1 and integrins. Besides SIRP α, SIRP β1 and SIRP γ have also been identified in humans. Both of SIRP β1 and SIRP γ consist of three Ig-like loops in their extracellular domains. SIRP β1 is characterized by a basic amino acid side chain in its transmembrane domain with a very short cytoplasmic region. This transmembrane region is indispensable for binding of DAP12 (DNAX activation protein). There is also a short cytoplasmic region in SIRP γ, which was established to play an important role in T-cell *trans*-endothelial migration [129]. SIRP δ has only one domain and has not yet been found any obvious means of membrane attachment [18].

CD47 belongs to immunoglobulin superfamily having a short C- terminal intracellular tail, a five-transmembrane- domain and N- terminal IgV extracellular domain [[1], [2], [3], [4], [5], [6], [7]]. It was demonstrated that CD47 plays an integral role in various immune responses as well as various pathophysiological processes by sending a potent “don't eat me”anti-phagocytic signal [8]. TSP-1 (thrombospondin-1) is another high-affinity CD47 ligand, which regulates cellular signaling pathway such as redox control, inflammation, and self-renewal [9,10]. Furthermore, CD47 also interacts with some typical transmembrane integrins including the well-characterized integrin α_V_β_3_ and α2β1 [6] **(***see* Fig. 1).

CD47 is allocated with multiple fundamental cellular functions, like cell migration, apoptosis and axon development [[11], [12], [13], [14], [15], [16], [17]]. It is widely expressed on the surfaces of normal cells and many different types of cancer [18]. In general, SIRPα is considered as the most important ligand of CD47 and targeting CD47-SIRPα axis is regarded as a novel strategy in the treatment of cancer.

Cancer immunotherapy has the inspiration to revolutionize the treatment of noncancerous diseases by utilizing the immune system's capabilities to target specific cells or molecules. Principles and strategies employed in CD47-related cancer immunotherapy can be appropriately adapted and applied to other medical conditions. The impressive success and advancements in CD47 cancer immunotherapy have generated hope and opened new avenues for innovative approaches in treating a wide range of noncancerous ailments. It is necessary to summarize the treatment of CD47 in non-tumor diseases, and explore what kind of inspiration can be obtained from tumor treatment and how to avoid problems in tumor treatment.

This paper aims to explore the role of CD47 and briefly outline it signaling pathways. Additionally, we will focus on the latest advancements in CD47-based immunotherapy for non-neoplastic diseases and propose several strategies to address the current challenges faced in CD47-based immunotherapy. Furthermore, we will discuss the potential clinical application of the CD47-SIRPα axis as a therapeutic target while also acknowledging the obstacles and potential directions for future research.

## 2 Ligands of CD47

CD47 has several known ligands, including signal regulatory protein alpha (SIRPα), thrombospondin-1 (TSP-1), thrombospondin-2 (TSP-2), and signal regulatory protein beta-1 (SIRPβ1).The most investigated binding partner of CD47 is SIRPα, which is highly expressed in neurons and in a subset of myeloid hematopoietic cells such as dendritic cells (DCs) and macrophages [19]. SIRPα is a transmembrane protein comprised of four ITIMs (immunoreceptor tyrosine-based inhibition motif) in the cytoplasmic region and three immunoglobulin (Ig)-like domains in extracellular region [20]. ITIMs provides the binding sites for the Src homology-2 (SH2)-domain-containing protein tyrosine phosphatases SHP-2 and after that bind to the cytoplasmic region of SIRPα to mediate the subsequent immune responses. The NH_2_-terminal V-like domain is critical for binding to CD47 [18,21,22].

While the SIRPβ1 receptor does not engage with CD47, the interaction between CD47 and SIRPγ, found in human T cells and NK cells but not in rodents, has been demonstrated to facilitate T cell adhesion to antigen-presenting cells (APCs). This adhesion process leads to T cell activation and proliferation [18,23]. Therefore, a potential ideal approach for cancer immunotherapy could involve inhibiting the interaction between SIRPα and CD47 while maintaining the binding between SIRPγ and CD47 [24].

TSP-1 is a large extracellular matrix protein that serves also as a ligand for CD47. When TSP-1 binds to CD47, it can regulate cellular processes such as cell migration, proliferation, and adhesion. This interaction is essential in various physiological and pathological contexts, including immune responses and tissue remodeling [10,25]. TSP-2 shares homology in its C-terminus to TSP1 and binds CD47with less affinity. Its interaction with CD47 can influence cellular behavior and functions, such as cell adhesion and signaling pathways [26]. But the affinity ability would increase in visceral and gonadal fat of diet- and genetic-driven mice with obesity [27].

In addition, CD47 can interact with several integrins, which are a family of cell surface receptors involved in cell adhesion and signaling. It was demonstrated that CD47 associate in cis with β1, β3, 4N1K, and Mac-1 (αMβ2, CD11b/CD18) integrins [6,28,29]. The interaction between CD47 and integrins plays a crucial role in various cellular processes, including immune responses, cell migration, and tissue development. Recently, CD47 and several integrins have been identified as ubiquitous and abundant membrane components of extracellular vesicles from a variety of cell types [30]. They also have implications in various physiological and pathological contexts. Further research on the interactions can provide valuable insights into cellular functions and potential therapeutic strategies for various diseases.

## 3 CD47 in immunotherapy

In cancer immunotherapy, CD47 inhibition has gained attention as a potential therapeutic strategy. By blocking CD47, researchers aim to enable the immune system to recognize and attack cancer cells more effectively. When CD47 is inhibited, macrophages can recognize the cancer cells as foreign and phagocytose them, helping to eliminate the tumor. Several experimental therapies that target CD47 are being explored, including monoclonal antibodies and other agents that block the CD47 signal. Such approaches aim to boost the body's immune response against cancer, offering promising possibilities in the field of cancer immunotherapy.

Cancer immunotherapy has the potential to inspire the treatment of noncancerous diseases through its focus on harnessing the power of the immune system to target specific cells or molecules. The principles and strategies employed in CD47-related cancer immunotherapy can be adapted and applied to other diseases. However, it's important to note that while cancer immunotherapy provides valuable insights and strategies, each disease has unique characteristics and challenges. The translation of cancer immunotherapy principles to noncancerous diseases requires careful research, testing, and clinical trials to ensure safety and efficacy. The success and advancements in CD47 cancer immunotherapy have sparked optimism and paved the way for innovative approaches in treating a wide range of noncancerous conditions.

### 3.1 CD47 and atherosclerosis

Atherosclerosis is a specific type of arteriosclerosis which is responsible for several important adverse vascular events, like coronary artery disease, myocardial infraction, stroke and peripheral artery disease [31]. The biological and clinical characteristics of atherosclerosis include deviant immune reaction, chronic inflammation, and dysfunctional lipid metabolism [32]. The manner in which CD47 contribute to arteriosclerosis is still a subject of considerable debate. *Kojima* et al. have identified that CD47 is consistently upregulated in atherosclerotic plaque of symptomatic stroke patients in contrast to asymptomatic patients [33]. Furthermore, it has been shown that CD47 expression level increases continuously in the process of atherosclerosis development and anti-CD47 antibody reduce vascular inflammation in the carotid arteries of participants [[33], [34], [35], [36], [37]]. Bioinformatics approach proves that there is a strong association between CD47 and both TNFR1 (type I TNF receptor) and TNF-α [33]. Additionally, blocking CD47 was found to decrease the activity of genes associated with the macrophage response to IL-1 and IFN-γ, resulting in a notable decrease in atherosclerotic inflammation observed through PET-CT imaging of mouse models [38]. Moreover, CD47 has been proposed to hinder macrophages from eliminating opsonized targets, such as opsonized clonal smooth muscle cells which were believed to be the primary contributors to atherosclerotic plaques [39]. By administration of CD47-blocking antibodies, the atheroprone mice would develop significantly smaller atherosclerotic plaques compared to IgG controls [33].

**Fig. 2:**
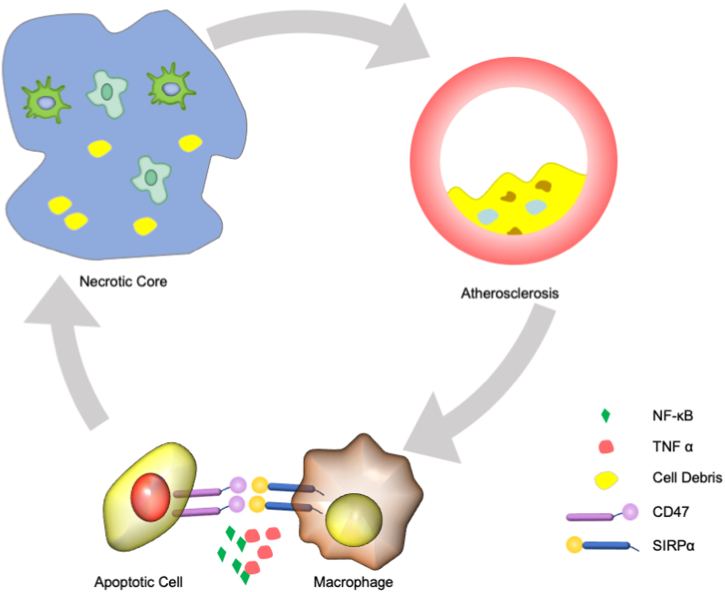
CD47 expression is promoted by TNF-α via NF-κB1 and gets involved in the pathological process of atherosclerosis CD47 was upregulated in both human coronary and carotid atherosclerotic vessels, presumably via a TNF-α and NFκB1-dependent process; Interaction between CD47 on apoptotic cells and SIRPα on macrophages promotes anti-phagocytic signal and finally induces defective efferocytosis signaling, which further reduces the edibility of cells within necrotic core; Finally, apoptotic cells in the growing plaque turns impoverished substrates for phagocytic cells and the residue become trivial necrotic and would release more pro-inflammatory stimuli, resulting in a positive feedback cycle. Anti-CD47 antibodies can stimulate efferocytosis in TNF-α -treated cells and dramatically countered the buildup of arterial plaque and made it less vulnerable to rupture.

TNF-α, a pro-inflammatory cytokine associated with atherosclerosis development, promotes CD47 expression on the surface of apoptotic vascular smooth muscle cells [[40], [41], [42]]. In regard with previous studies, CD47 blockades were able to stimulate efferocytosis in TNF-α-treated cells [43,44]*.* In addition, TSP-1-CD47 interaction redundantly inhibits antagonism NO (nitric oxide)-cGMP-cGKI axis, which plays a pro-atherogenic role in atherosclerosis development [45] *(see* Fig. 2*)***.**

Research had previously proved that blocking CD47 activity rescues defective efferocytosis and decreases the atherosclerotic burden in mice and plaque inflammation in humans. In 2022，the group led by *Nicholas Leeper* demonstrated statins increase the ability of efferocytosis by downregulating CD47 on apoptotic cells [[46], [47], [48], [49]]. By inhibiting nuclear translocation of the inflammatory transcription factor NF-κB1 p50, atorvastatin reduces CD47 expression and the combination of atorvastatin and CD47–SIRPα blockade induces an additive effect on efferocytosis and atherosclerotic plaque size and inflammation [46].

Since atherosclerosis is the basis of most cardiovascular diseases, targeting CD47 signaling may also provide a promising approach to the prevention and treatment of cardiovascular diseases. Various strategies are being developed to inhibit CD47, including RNA interference and antibody blockade technology [50]. In study by *Stanley J. S*, they demonstrated that stents functionalized with pepCD47 effectively prevented fibrin and platelet thrombus deposition. Additionally, they inhibited inflammatory cell attachment and led to a 30 % reduction in restenosis. It was concluded that CD47-modified stent surfaces have the potential to mitigate platelet and inflammatory cell attachment, effectively disrupting the pathophysiology of in-stent restenosis [51].

### 3.2 CD47 and neurological disorders

#### 3.2.1 CD47 and cell development in CNS

As the resident macrophage in CNS, microglia acts in close contact with neurons, astrocytes and oligodendrocytes [52]. Viable neurons evade being cleared off by displaying the anti-phagocytic receptor CD47 which binds to SIRPα on microglia [53]. Otherwise, live neurons may be phagocytosed by microglia via “eat me” C1q-CR3 signal during pathological states [54]. A balance between pro-phagocytic and anti-phagocytic signals plays an important role in maintaining homeostasis in the central nervous system [55].

**Fig. 3:**
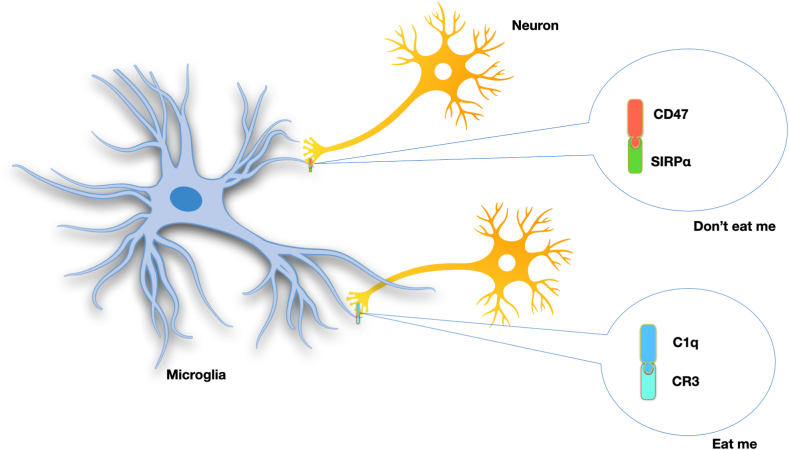
Opposing signals in synaptic pruning. CD47 expressed on neurons interacts with SIRPα on microglia, which discourage the immune cells from digesting the synaptic terminals [17]; In addition, unnecessary synaptic connections that are not used to transmit signals are removed by a process called pruning, which is partly carried out via interaction between the neuronal release of C1q, the initiator of the classical complement cascade and the CR3(complement receptor 3,the microglial phagocytic receptor that detects C3b) [54].

The role of CD47 in neuronal development is an active area of research as it may be a target in treating a group of neurological disorders. In neurogenesis, CD47 was found to plays a role in improving dendritic outgrowth, up-regulation of synaptic proteins, and glutamate release through the MAPK (mitogen-activated protein kinase)-targeted) pathway [56]. A recent study identified that CD47 facilitates the development of dendrites and axons in hippocampal neurons in a manner which dependent on activation of Cdc42 and Rac [57]. In 2018, *Lehrman* et al. showed that CD47-SIRPα signaling inhibits redundant microglial phagocytosis during developmental synaptic pruning, which provides evidence that synaptic protection is essential to ensure normal circuit development [17]**.** CD47 can also be regarded as the first example of a protective molecule that inhibits inappropriate pruning in the visual system [17] *(see* Fig. 3.

#### 3.2.2 CD47 and brain hemorrhage

Traumatic brain injury (TBI ), cerebrovascular disorders and Alzheimer's disease are the three main causes of disability from neurological diseases and collectively account for the largest global burden of disability [58]. CD47 is broadly involved in neuronal cell death, neuro-inflammation, and progression of acute ischemic brain injury [59]. In the pathological cascades following TBI, CD47 plays a crucial role in vascular pathophysiology after brain injury, especially in the anti-angiogenesis effect via TSP-1-CD47 interaction [60,61]. The utilization of CD47 blockades may provide a platform for the further development in reducing inflammatory injury and improving vascular remodeling in TBI. In another pig model of intracerebral hemorrhage, *Zhou* and colleagues identified that brain tissue displayed an increased percentage of CD47 expression in both white and gray matter [62]. Similarly, deletion of CD47 also played a protective role in spinal cord injury model which were characterized by improved penumbral vascularity, enhanced white matter sparing and decreased inflammation [63]. Recently, there has been considerable interest in neurosurgery field targeting CD47. For example, injection of CD47-deficient blood or CD47 antibody administration lead to faster hematoma clearance and reduced secondary brain injury after intracerebral hemorrhage [64,65]. In future, CD47 blocking therapy may become potential adjunct therapy in intracranial hematoma or TBI after availability of more data.

#### 3.2.3 CD47 and cerebral ischemia

Ischemic stroke shares lots of pathological mechanisms with TBI. In the context of acute stroke, CD47 and its ligands are upregulated in brain neurons and endothelial cells [66]. Over-activation of CD47 triggers cell death pathway in a wide array of cell types such as immune cells and cancer cells [67]. Similarly, this situation also appears to occur in neurons and endothelial cells, leading to multiple deleterious effects in the brain [68,69]. Further research demonstrated that CD47 promotes MMP-9 (matrix metalloproteinase-9) and VEGR (Vascular endothelial growth factor) upregulation after stroke, which contributes to increased inflammatory cell infiltration and aggravated neuro-inflammation in the ischemic brain [70]. These research opens new horizons in the potential therapeutic applications of targeting CD47 to attenuate neuronal damage in cerebral ischemia.

#### 3.2.4 CD47 and Alzheimer's disease

Alzheimer's disease is a slowly progressive neurodegenerative disease characterized by accumulation of abnormally folded Aβ and tau proteins in amyloid plaques and neuronal tangles [71,72]. Microglia are emerging as key players in Alzheimer's disease since it plays a direct role in ‘neuro’ degeneration by promoting phagocytosis of neuronal, in particular, synaptic structures [73]. CD47 in Alzheimer's disease has been shown that CD47 facilitates Aβ oligomers internalization by microglia [74,75]. In another recent study, CD47 has been exploited as part of a long-circulating delivery drug (CRT-CD47-NP-Nec-1s) in the treatment of Alzheimer's disease [76]. Clearly, microglia-targeted drug delivery with CD47 participation is very helpful for Alzheimer's disease treatment.

Recently, isoQC (Glutaminyl-peptide cyclotransferase-like protein) has been identified as a key regulator of the CD47-SIRPα checkpoint and is critical for the pyroglutamylation of CD47 at its SIRPα binding site [77]. Inhibition of isoQC blocks the interaction between CD47 and SIRPα, leading to constrained tumor growth [78,79]. Importantly, IsoQC resides in the Golgi complex, which is absent in RBCs; therefore, targeting isoQC can overcome the adverse side effects of traditional CD47 inhibitors [[80], [81], [82], [83]]. Therapeutic potential of QC inhibitors has been explored and shown promising new promising therapeutic avenues. PQ-912 (also known as Varoglutamstat) has been applied in clinical trials and has completed a phase 2a trial in AD [[84], [85], [86], [87]] [[84], [85], [86], [87]] [[84], [85], [86], [87]]. The phase 2 trial (NCT02389413) called SAPHIR, showed that treated patients with PQ912 had an improvement in memory and PQ912 was safe, with a frequency of side effects similar to those seen in a placebo group. These positive results led investigators to start a Phase 2b trial (NCT03919162) to evaluate the safety and efficacy of PQ192 in patients with early AD.

Taken together, these discoveries encourage further testing and engineering of CD47 for the clinical management of different neurological disorders. Even in the early stage understanding of its diverse function and potentiality, small molecules targeting CD47-SIRPα as a potential target deserves more investigation.

### 3.3 CD47 and muscle stem cells

It was demonstrated that CD47 signaling plays a role in promoting the proliferation of young muscle stem cells (MuSCs) in the presence of hypertrophy induced by mechanical stress. This resulted in the accumulation of myonuclei within the muscle cells [88]. Present research by *Helen* M. B et al. indicates that unlike young MuSCs, the cell surface of aged MuSCs exhibits a significant increase in CD47 expression [89]. This upregulation is attributed to alternative polyadenylation, which is a consequence of elevated U1 snRNA expression. Therefore, CD47 expression can be used to distinguish functionally and molecularly distinct aged muscle stem cell subsets. As individuals age, TSP-1 has been observed to accumulate in various tissues, such as skeletal and cardiac muscle [90]. Additionally, its expression in skeletal muscle has been found to decrease in response to exercise [[90], [91], [92]] [[90], [91], [92]] [[90], [91], [92]]. Additional research is needed to explore the potential of immunotherapies that target TSP-1/CD47 signaling in peripheral tissues, such as muscle. Such therapies could potentially activate MuSCs and enhance stem cell proliferation, offering a possible strategy to counteract muscle wasting that often occurs in cancer patients.

### 3.4 CD47 and diabetes

The relationship between CD47 and diabetes is an area of active research. While an understanding of how CD47 affects diabetes is continually evolving, there are several aspects worth discussing.

Type 1 diabetes is an autoimmune disease characterized by the destruction of insulin-producing β cells in the pancreas. β cells in the pancreas play a crucial role in the production and regulation of insulin, which is essential for maintaining normal blood sugar levels. The immune system mistakenly targets these cells, leading to insulin deficiency. There is a significant need for therapeutic agents that enhance insulin secretion and improve insulin sensitivity in the treatment of diabetes. In this context, CD47 expression on β cells may have implications. Research suggests that dysregulated CD47 signaling could contribute to the failure of immune tolerance, leading to the destruction of β cells by immune cells, such as T cells and macrophages. In a previously conducted LC-MS/MS-based quantitative proteomic screen, researchers investigated the time-resolved phospho-proteome of pancreatic cells in NOD mice exposed to glucose to promote insulin secretion. The findings revealed a noticeable trend of elevated CD47 expression, progressing from nondiabetic to overtly diabetic mice [93]. In subsequent studies, blocking CD47 showed the ability to delay the onset of overt diabetes in NOD mice, with minimal variations in insulitis scores compared to mice treated with an isotype control. This finding suggests that the improvement in insulin secretion, rather than alterations in inflammation, primarily contributed to the enhancement of glucose homeostasis. These results indicate that CD47 receptor antagonism could potentially be clinically advantageous in prolonging the honeymoon period in new-onset type 1 diabetes patients by boosting insulin secretion. Present data has also demonstrated that CD47 receptor signaling inhibits insulin release from β-cells and that it can be pharmacologically exploited to boost insulin secretion [94].

Given the involvement of CD47 in diabetes pathogenesis, targeting CD47 has emerged as a potential therapeutic strategy. However, further research is required to fully understand the underlying mechanisms and to explore the potential of CD47-targeted immunotherapies in the treatment of diabetes.

### 3.5 CD47 and other autoimmune diseases

CD47-SIRPα has been proposed as a regulator in the development of T cell-mediated autoimmune pathogenesis, including multiple sclerosis (MS), autoimmune hemolytic anemia (AIHA) and autoimmune diabetes [19,95].

MS is an unpredictable autoimmune disease of the central nervous system [96]. It is characterized by myelin sheath degradation and affects 2 million people worldwide [96]. The “don't eat me” signaling protein CD47 is found downregulated in MS lesions [97]. The main reason for this may be related to its role as a shared target of eight upregulated miRNAs, including miR-155, miR-34a and miR-326 [98]. This reduced CD47-SIRPα promotes phagocytosis of compact myelin and splenocytes. Modulating CD47 has a potential role for therapy in MS deserves further investigation.

AIHA develops when there is production of antibodies directed against self-red blood cells [99]. Specifically, the finding that the lack of CD47 on RBCs is responsible for the severity of AIHA in CD47/NOD mice led researchers to speculate that the CD47 mimetics might be an alternative to treatments aimed at eliminating the accelerated clearance system [100].

## 4 Novel therapeutic strategies of CD47-targeting antibodies

**Fig. 4:**
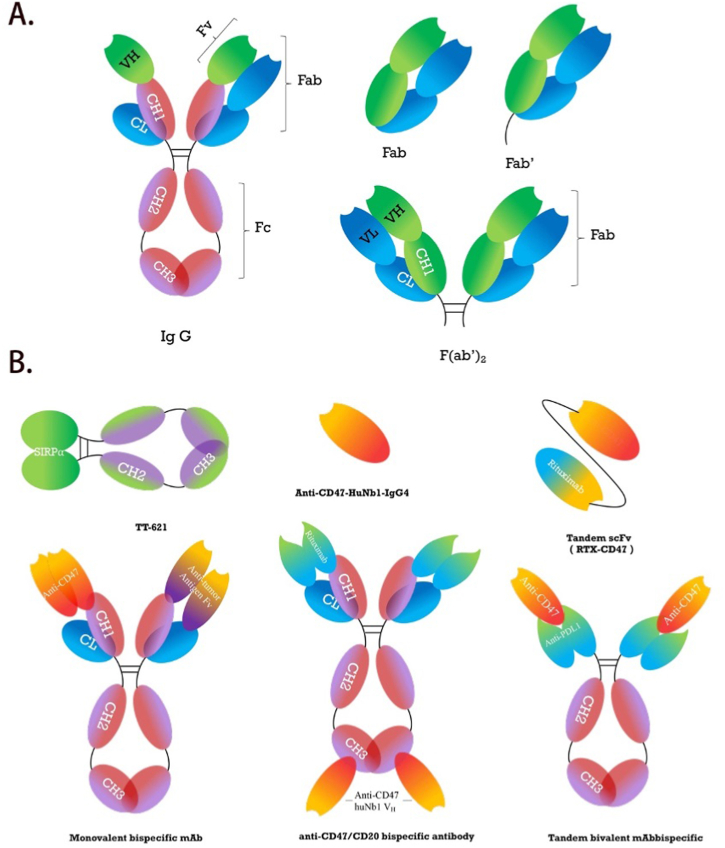
A. Structures of IgG antibody and its variants. IgG antibody are composed of four heavy chains and two light chains. Fab' contains disulfide bridge thiols that can be distinguished from Fab while F(ab')_2_ fragment consists of two Fab’ regions connected at hinge region [101,102]. B. Structures of therapeutic antibodies involved in CD47-SIRPα interaction. i. TTI-621 (SIRPαFc) is a novel immunotherapeutic consisting of the CD47 binding domain of human SIRPα linked to the Fc region of human IgG1 [105]. ii. HuNb1-IgG4 was developed with high affinity and specificity and effectively enhanced macrophage-mediated phagocytosis of tumor cells [108]. iii. RTX-CD47 is a novel construct that combines a CD20-targeting single-chain variable fragment (scFv) derived from rituximab with a CD47-blocking scFv, creating a tandem fusion antibody [130]. iv. Monovalent antibodies overcome limitations of antibody bivalency for targets impacted by antibody crosslinking. For example, TG-1801 is a bispecific agent specifically designed to engage both CD19 and CD47 simultaneously on the surface of tumor cells, with one arm possessing high-affinity for CD19 and the other arm blocking CD47 [131,132]. v. Bispecific antibodies (BsAbs) that co-target CD47 and CD20 was demonstrated with reduced affinity for CD47 relative to the parental antibody, but this BsAbs retain strong binding to CD20 [133]. vi. IBI-322 is a different bispecific antibody (BsAb) designed to target both PD-L1 and CD47, and it is currently undergoing phase 1 trials for the treatment of advanced tumors [134].

As shown in Fig. 4, IgG antibody are composed of four heavy chains and two light chains. Fab' contains disulfide bridge thiols that can be distinguished from Fab while F(ab')_2_ fragment consists of two Fab’ regions connected at hinge region [101,102]. These three fragments can penetrate tissues more efficiently and can easily be cleared because of their small size. In addition, they don't interfere with anti-Fc mediated antibody detection, thereby were used to testify if CD47-antibodies functions through Fc-dependent mechanisms [103].

Theoretically speaking，SIRPα might have been more ideal since it is narrowly expressed on health cells compared with CD47 [104]. TT-621 consists of the N-terminal domain of human SIRPα linked to a human IgG1 Fc region and was demonstrated with broad antitumor activity [105]. However, blocking SIRPα may affect the functions of other SIRP family members [106]. Therefore, we place more emphasis on these antibodies against CD47. Firstly, nanobodies (Nbs) have been regarded as an ideal therapeutic reagents due to their small size, high affinity and stability [107]. Recently, a novel anti-CD47 Nb fusion protein HuNb1-IgG4，a Nb-based human heavy chain antibody combined with advantages of Nbs and human Fc domains, has demonstrated the potency *in vitro* against ovarian tumor and lymphoma [108]. Taking advantage of this, an anti-CD47/CD20 bispecific antibody combining HuNb1-IgG4 and variable domain from Rituximab was further explored and it exhibited increased efficacy in mouse xenograft B cell lymphoma models [108]. Secondly, RTX-CD47, a CD20-targeting scFv antibody fragment fused in tandem to a CD47-blocking scFv, promotes selective removal of CD47^+^CD20^+^ cells through phagocytosis. Absence of Fc domain allows it to avoid triggering of FcR-mediated immunological processes [109]. Thirdly, CD47/PD-L1 dual-targeting bispecific antibody was constructed using h4c1 and variable domains from anti-PD-L1 [110]. It also displays substantial tumor suppression efficacy with limited hemagglutination, paving the way for next-generation anti-CD47 antibodies to improve tumor checkpoint therapy [110].

## 5 Discussion

CD47 has been extensively studied and is known to play a critical role in immune evasion by cancer cells. It acts as a "don't eat me" signal, preventing immune cells from engulfing and eliminating cancer cells. With the promising potentials of anti-CD47 blockades in cancer immunotherapy, there is ongoing interest in expanding this field in the treatment of non-neoplastic diseases.

CD47 blockade as a therapeutic strategy in non-neoplastic diseases is an emerging field of research with promising preclinical results. The blockade has shown potential in modulating immune responses, reducing inflammation, and promoting tissue repair in various non-neoplastic conditions. However, further studies and clinical trials are needed to fully evaluate its safety, efficacy, and potential applications in non-neoplastic diseases.

The primary challenge of CD47 blockade as a therapeutic strategy lies in achieving optimal selectivity, avoiding off-target effects, and overcoming potential resistance to maximize its therapeutic efficacy in various disease contexts.

Firstly, during the investigation of the CD47-SIRPα axis, the precise molecular mechanism underlying the CD47-SIRPα signaling pathway remains elusive. As is known, CD47-SIRPα interactions result in the phosphorylation of two tyrosine residues in the intracellular ITIM, which subsequently recruits and activates SHP1 and SHP2. However, the signaling pathway by which SHP-1/2 influences phagocytosis is not yet definitively understood. Activation of SHP1 has been shown to reduce the phosphorylation of ITAM-like targets and compete with Syk phosphatase at the interface, ultimately modulating phagocytosis. Additionally, research by *Morrissey* et al. suggests that the CD47-SIRPα axis suppresses macrophage spreading and phagocytosis by inhibiting integrin activation rather than altering Syk recruitment [111]. This signaling cascade leads to the dephosphorylation of myosin IIA and inhibition of cytoskeleton rearrangement, a critical step for phagocytosis. However, the studies conducted so far have a research bias towards simulating macrophage phagocytosis of tumor cells, and the specific signaling pathway between SHP-1 and myosin IIA is still not fully characterized. Further investigations are needed to gain a comprehensive understanding of these interactions.

Secondly, in the stage of CD47 blockade clinical application, a wide range of toxicities with varying severity have been observed, largely dependent on the specific antibody used. The most frequently encountered toxicity was the anticipated on-target anemia, which arises as a pharmacodynamic effect resulting from the blockade of CD47 [112,113]. CD47, as a marker of self, is highly expressed on RBCs. RBCs lacking CD47 are rapidly cleared from the bloodstream, triggering the physiological clearance of aging RBCs [114]. In clinical trials of CD47 blockade, anemia was a common toxicity observed, with a drop in hemoglobin (Hb) levels after dosing requiring RBC transfusion in patients. Strategies such as priming doses and next-generation anti-CD47 antibodies with reduced binding to RBCs aim to mitigate treatment-related anemia and minimize on-target adverse effects [112,113,115]. A potential approach to address this issue is to reduce the binding of CD47 to RBCs, which could potentially ameliorate anemia and improve the safety profile of CD47-targeting treatments. By achieving a more selective and refined CD47 blockade, the unwanted effects on RBCs could be minimized [2,116].

In addition, new generation CD47 antibodies have been developed in an effort to overcome the limitations of CD47-targeting therapies and improve treatment efficacy. These antibodies efficiently target tumor cells while causing minimal adverse effects on RBCs, thus avoiding severe anemia. Additionally, several well-designed BsAbs have been created, such as IMM2902 for CD47-Her2, IMM0306 for CD47-D20, and IBI322 for CD47-PD-L1, which effectively inhibit the CD47-SIRPα signal and enhance tumor cell phagocytosis without significant impact on RBCs [[117], [118], [119], [120]] [[117], [118], [119], [120]] [[117], [118], [119], [120]].

Thirdly, in the development of anti-CD47 bsAbs, another challenge arises from the widespread expression of CD47 on normal tissues, leading to an "antigen sink" effect that hinders the effective binding of therapeutic antibodies with targeted tumor cells *in vivo*. This may necessitate high initial doses and repeated administration of medication to achieve efficient CD47 blockade. In contrast, SIRP has a less extensive distribution than CD47, potentially resulting in a stronger blocking effect and fewer side effects in targeted therapy [121]. However, the possible cross-reactivity between different SIRP family members, such as SIRPβ and SIRPγ, and its implications remain unclear [122].

One approach to address this challenge is to reduce the affinity of bsAbs for CD47 while maintaining strong blocking of the CD47-SIRPα interaction and increasing the affinity for binding to another tumor antigen [123]. Additionally, the use of anti-SIRPα 1H9 can help overcome the antigen sink effect, potentially requiring a lower medication dosage, although the issue of lacking a monotherapy effect needs consideration [122]. To circumvent the limitations associated with the antigen sink effect, future studies should focus on directly targeting CD47 and its ligands on tumor cells to enhance the specificity and efficacy of CD47-targeting therapies.

Fourthly, therapeutic failures in cancer immunotherapy are often attributed to the development of resistant cancer cell clones and tumor heterogeneity, which can compromise the effectiveness of single targeted treatments. Resistance arises due to the adaptability and flexibility of cancer signaling networks, driven by intrinsic and extrinsic factors [124,125]. Intrinsic mechanisms include alterations in tumor cell signaling pathways, changes in antitumor immune response pathways, and the development of an immunosuppressive microenvironment. Extrinsic factors, such as the tumor microenvironment and host-related factors, can also contribute to tumor proliferation and resistance to immune checkpoint inhibitors. Additionally, various factors, including changes in the tumor microenvironment, drug inactivation, reduced drug absorption, increased drug release from tumor cells, activation of tumor cell survival pathways, and epigenetic changes, can lead to drug resistance. Moreover, the choice of tumor model used in preclinical research may influence the overall response to anti-CD47 therapy, emphasizing the importance of considering tumor heterogeneity and complexity in therapeutic development [126].

Fifthly, when designing clinical studies about the treatment of non-neoplastic disease, it is crucial to consider the functions and characteristics of immune cells, such as tumor-specific cytotoxic T lymphocytes, as endpoints for immunotherapy [127]. Unlike traditional therapies that directly attack tumor cells, non-neoplastic disease immunotherapy involves activating the immune system, which can result in delayed responses during clinical trials. Therefore, it is essential to carefully select appropriate endpoints in anti-CD47 clinical trials to accurately assess the efficacy of immunotherapy [128]. The development of immunological memory in response to various immunotherapies necessitates the inclusion of endpoints to measure long-term disease-free survival. Developing anti-CD47 therapies requires careful consideration of how to minimize or avoid harm to normal cells while achieving effective effects.

In summary, the applications of CD47-SIRP-based therapy in the treatment of non-neoplastic diseases are still in early stage. Limit data has demonstrated promising promising results, and it is highly hopeful that some candidate agents will emerge and make into clinical application to meet the urgent needs of non-neoplastic diseases patients. The current use of CD47-SIRP-based therapy faces multiple challenges, but it also presents several potential opportunities. To enhance existing strategies, further research is needed to identify effective delivery methods. Although single monotherapy approaches targeting this axis have shown limited efficacy, we propose exploring various combinatorial therapies that combine these monotherapies with standard-of-care agents. Such an approach may hold promise in overcoming the limitations and improving the effectiveness of CD47-SIRP-based therapy in the treatment of non-neoplastic diseases.

## Ethical Approval

Not applicable.

## Funding

This work was funded by the 10.13039/501100007129Shandong Province Natural Science Foundation grants ZR2022QH372.

## Data availability statement

No data was used for the research described in the article.

## CRediT authorship contribution statement

**Chao Wang:** Writing – original draft. **Ying Feng:** Data curation, Writing – original draft. **Deepali Patel:** Writing – original draft. **Hongwei Xie:** Conceptualization, Investigation, Software. **Yaqing Lv:** Investigation. **Hai Zhao:** Writing – review & editing, Validation, Supervision, Software, Resources, Conceptualization.

## Declaration of competing interest

The authors declare that they have no known competing financial interests or personal relationships that could have appeared to influence the work reported in this paper.
